# Beyond Invisible: Confronting the Policy and Practice Void in Eating Disorder Prevention and Treatment for Indigenous Peoples in Canada

**DOI:** 10.3390/bs16091558

**Published:** 2026-09-02

**Authors:** Katherine Fredrickson, Amelia Austin, Maureen Plante, Kienan Williams, Gina Dimitropoulos, Carolyn Melro

**Affiliations:** 1 Children’s Hospital of Eastern Ontario Research Institute, Ottawa, ON K1H 5B2, Canada; kfred063@uottawa.ca; 2 Faculty of Education, University of Ottawa, Ottawa, ON K1N 6N7, Canada; 3 Department of Clinical Psychology, University of Edinburgh, Edinburgh EH8 9JZ, UK; amelia.austin1@ucalgary.ca; 4 Department of Psychology, Glasgow Caledonian University, Glasgow G4 0BA, UK; 5 Faculty of Education, University of Alberta, Edmonton, AB T6G 2J5, Canada; msplante@ualberta.ca; 6 Primary Care Alberta, Calgary, AB T5J 1S6, Canada; kienan.williams@ucalgary.ca; 7 Faculty of Social Work, University of Calgary, Calgary, AB T2N 1N4, Canada; gdimit@ucalgary.ca; 8 Mathison Centre for Mental Health Research & Education, University of Calgary, Calgary, AB T2N 5A1, Canada; 9 Department of Psychiatry, University of Calgary, Calgary, AB T2N 1N4, Canada; 10 Faculty of Medicine, University of Ottawa, Ottawa, ON K1H 8M5, Canada

**Keywords:** anorexia nervosa, binge eating, bulimia nervosa, First Nations, Indigenous, Inuit, Métis, eating disorders

## Abstract

Indigenous populations internationally experience eating disorders (EDs) at similar or higher rates than colonizing majority populations. In Canada, emerging evidence suggests that this is also true among First Nations, Métis, and Inuit (FNMI) populations. However, most existing recommendations and best practices for ED prevention, early intervention, and treatment do not consider unique contextual and cultural factors that are relevant to FNMI Peoples, such as the effects of historical and ongoing colonialism. This scoping review aimed to map policy-related documents on the prevention, early intervention, and treatment of EDs among FNMI Peoples. We searched academic databases and targeted websites for ED policies, reports, toolkits, guidelines, and fact sheets. Targeted searches included the websites of Indigenous health authorities (e.g., National Collaborating Centre for Indigenous Health) and community websites, ED-specific organizations (e.g., National Eating Disorder Information Centre), and health professional associations (e.g., Canadian Psychological Association). We found only two relevant documents: one commentary serving as a call to action and one education resource for people experiencing EDs or disordered eating and their family members. Both documents were led by Indigenous authors with lived experience of EDs and were published by the National Eating Disorder Information Centre. In the absence of wider guidance on prevention, early intervention, and treatment among FNMI populations, health professionals may face challenges when providing effective and culturally responsive care. This review illustrates the infancy of the policy landscape related to the prevention, early intervention, and treatment of EDs for FNMI populations in Canada.

## 1. Introduction

Eating disorders (EDs) are serious and multifaceted public health concerns that affect physical, psychological, and social functioning (Treasure et al., 2020), and spiritual connection (Plante et al., 2026), with a peak onset in adolescence and emerging adulthood (Solmi et al., 2022). While an estimated 2.9 million Canadians are affected by an ED (National Initiative for Eating Disorders, 2025), the true prevalence is likely higher given the absence of a federal health surveillance framework and underreporting among equity groups with EDs (Raffoul et al., 2025). Since the COVID-19 pandemic, healthcare settings across Canada have seen marked increases in ED presentations (Katzman et al., 2025; Milliren et al., 2023; Toulany et al., 2025).

While national prevalence estimates of EDs in First Nations, Métis, and Inuit (FNMI) populations in Canada are not yet widely available, evidence of EDs and disordered eating affecting Indigenous populations from countries with similar colonial histories (e.g., Australia, Aotearoa New Zealand, United States) indicates that Indigenous Peoples experience similar or higher rates in comparison to the colonizing majority ethnic group (Burt et al., 2020; Masters et al., 2025; Mitchison et al., 2023; Pettie et al., 2026; Williams-Ridgway et al., 2025). Data within Canada, though limited, point to similar trends: one specialist ED program in Thunder Bay reported that 21% of all youth referrals were FNMI (Mohammed et al., 2026). Despite this, existing recommendations and best practice guidance for ED prevention, early intervention, and treatment in Canada do not widely recognize factors in the etiology, maintenance, and recovery of EDs that may be uniquely important to FNMI populations.

FNMI Peoples’ experiences of food, body, and wellness are shaped by historical and ongoing colonial histories and intergenerational trauma (Neufeld et al., 2020). These include practices of forced relocation, rationing (Daschuk, 2013), malnutrition experiments, and residential schooling (Mosby, 2013), disrupting traditional food systems and embedding patterns of deprivation and shame around food that persist today. For example, food insecurity has been strongly linked to ED behaviors (Laboe et al., 2023; Trompeter et al., 2025), and is elevated in First Nations (Batal et al., 2021), Métis (Perchotte et al., 2025), and Inuit (Inuit Tapiriit Kanatami, 2018) populations in Canada. Repeatedly, the emphasis on traditional food and practices has been highlighted as a positive and impactful contribution to FNMI health (Desmarais & Wittman, 2014), yet policies have been used to control FNMI through food deprivation and starvation (Burnett et al., 2016; Mosby, 2013).

Acknowledging the trauma associated with healthcare practices is critical when determining the way forward with FNMI Peoples. The Truth and Reconciliation Commission of Canada (TRCC) presented Calls to Action urging the federal government to address the health disparities facing FNMI Peoples through recognition, consultation in policy development, increased targeted funding, and prioritizing FNMI representation within healthcare systems (TRCC, 2015). These imperatives are particularly relevant to EDs, where culturally sound care and distinctions-based approaches remain limited, and where seeking care can itself evoke trauma. A distinctions-based approach to care considers the similar but unique colonial experiences and cultures of FNMI Peoples. This underscores that effective ED policy must integrate dominant biomedical paradigms with FNMI knowledge and cultural practices toward strengths-based strategies that revitalize language and restore food sovereignty, cultural continuity, and collective wellness.

Guided by these historical and current factors, this scoping review aims to map publicly available policy-related documents on the prevention, early intervention, and treatment of EDs for FNMI populations in Canada. Within this work, we recognize that FNMI Peoples hold distinct histories, governance structures, and health systems, which we collectively define as taking a “distinctions-based” analytical approach. When possible, we aim to disaggregate policy-related documents among these groups, rather than describing a homogenous, pan-Indigenous identity in Canada.

## 2. Methods

This policy scoping review was guided by the framework developed by Arksey and O’Malley (2005). We adapted this based on the approach used in Gall et al.’s (2025) review on ED prevention and management for First Nations Peoples in Australia, which used an expanded conceptualization of policy to include a wide array of documents, including reports, toolkits, guidelines, strategy documents, annual reports, and frameworks.

Our study team interwove positionality considerations outlined by Chambers et al. (2018). We acknowledge our location within Western academic and hospital institutions and the limitations this brings to interpreting, synthesizing, and representing FNMI knowledges, emphasizing the need for humility, reflexivity, and accountability throughout this review. The authors of this paper are of mixed First Nations, Métis, *Status Indian*, and non-Indigenous identities. Our common goal is to share personal and professional experiences to improve mental health services and policy in the context of this paper by integrating First Nations and Métis knowledge systems to support care for patients and supportive others. We assume complex underpinnings and varied understandings of historical and ongoing effects of colonialism impacting FNMI in Canada. Throughout the process, we engaged in regular reflective circles to share, (un)learn, and challenge how we approach this work in a good way, ultimately strengthening the process and interpretation of this policy scoping review. A review protocol was not registered for this study.

### 2.1. Search Strategy

Upon conceptualization of the current review, we anticipated a similar gap to Gall et al.’s (2025) policy review. We conducted targeted searches of FNMI sources through respective searches of community health centers’, Indigenous-led health organizations’, and FNMI nations’ and organizations’ websites. Our broad search captured ED-focused organizations along with youth wellness organizations, mental health organizations, and health organizations at national, provincial, and territorial levels (Table 1). For each organization’s website, in February 2026, we navigated entire websites, opened all appropriately dated attachments, and used the search function for relevant information, while for FNMI community websites, throughout March and April 2026, we examined health and wellness tabs and used the websites’ search functions for mention of EDs when available. We relied on Indigenous Services Canada to provide First Nations and Inuit Community/Hamlet names to further hand search for community-controlled health center websites (Government of Canada, n.d.) and the Indigenous Health Links Interactive Map of health programs and services across Canada (NCCIH, n.d.). To expand our scope, we included peer-reviewed sources through a search of four academic databases (Bibliography of Indigenous Peoples in North America, CINAHL, PsycINFO, Medline). We developed search terms in collaboration with librarians, capturing terms related to EDs, as well as to FNMI populations, and informed by methodologies employed in previous research undertaken by members of the team (see Appendix A for search strategy). The search was conducted on 1 May 2026.

### 2.2. Eligibility Criteria

This policy scoping review adopts an expanded conceptualization of “policy” to include a broad assortment of concepts and associated documents (i.e., reports, toolkits, guidelines, strategy documents, annual reports, and frameworks), consistent with Gall et al. (2025). We took a broad view of policy, given the context in which we sought materials. We aimed not to exclude potential community resources based on a narrow, potentially Westernized definition of policy. Sources were included if they focused explicitly on First Nations, Métis, or Inuit, or addressed FNMI Peoples in ways that substantively informed ED-related policy, programming, or service and support considerations separate from a non-FNMI population. Although much work has emerged to include Black, Indigenous, and People of Color (BIPOC), we disaggregated to get a clear picture of FNMI policy and practice guidelines for EDs. Within this work related to FNMI populations, and despite our aim to take a distinctions-based approach, distinctions could only be made to the same level as the included publications.

This policy scoping review included publications that were or are active during an 11-year timeframe (2015–2026), to reflect the implementation of the TRC’s Calls to Action (TRCC, 2015) and the Canadian Psychological Association’s (Canadian Psychological Association & the Psychology Foundation of Canada, 2018) guidance on advancing Indigenous-led mental health approaches. When applicable, documents and policies implemented before 2015, but remaining active in 2015 and beyond, were also eligible. For example, publications would still be included if they were dated earlier than 2015 but remained active in that year. Eligible materials directly addressed EDs and were situated within the Canadian context, with specific relevance to FNMI populations.

### 2.3. Study Selection

Peer-reviewed records identified through database searches were imported into Covidence (*Covidence systematic review software*, 2026) for screening and management. Two team members (KF, MP) independently reviewed titles and abstracts in the Covidence platform, with conflicts resolved through discussion or consultation with a third reviewer when needed. Full-text screening was then completed using the same dual-reviewer process, using Covidence to track decisions, document reasons for exclusion, and maintain an audit trail consistent with PRISMA-ScR (Tricco et al., 2018) reporting standards. The PRISMA-ScR checklist is provided in Supplementary Materials File S1.

Our team undertook hand searches of relevant FNMI organizations’ (e.g., tribal councils, community-controlled health centers, government bodies, etc.), ED organizations’ (e.g., National Eating Disorder Information Centre, National Initiative for Eating Disorders, etc.), and health organizations’ (e.g., IYS hubs, mental health organizations, etc.) websites to identify policy documents not indexed in academic databases. Within Canada, a tribal council is a regional partnership of First Nations that works together to deliver shared services, and many councils provide community-based health programs such as primary care support, mental health, and culturally grounded wellness initiatives to strengthen the health of their member Nations. Given the initial focus on FNMI youth services and supports, we included hand searching of available websites for integrated youth services within Canada. Integrated youth services are a youth-focused service delivery model that addresses mental health and substance use, physical health, and related concerns among youth aged 12 to 25 years (Halsall et al., 2019), and their inclusion ensured that relevant policy-related documentation was captured for youth. Two team members (KF, CMM) completed the handsearching and entered all identified records into an Excel screening file, with a third reviewer available to consult when eligibility was uncertain. The grey literature screening and extraction is available in Supplementary Materials File S2. All materials identified through these searches were entered manually into the Excel screening spreadsheet, including document titles, source URLs, and preliminary eligibility notes. Only sources meeting all eligibility criteria were advanced to data extraction.

### 2.4. Data Extraction and Synthesis

A standardized data-charting extraction sheet was developed in Excel to capture key characteristics of each source, including year of publication, document title, authors/authoring organization (where applicable), author names (where applicable), document type, intended audience, and mentioned EDs, and references to cultural considerations/implementation and consultation of Indigenous stakeholder groups. The extracted articles are provided in Supplementary Materials File S2. Following data extraction and consistent scoping review methodology (Arksey & O’Malley, 2005), extracted data were synthesized descriptively.

## 3. Results

Across academic and grey literature sources, a total of 1191 articles and websites were screened to assess eligibility for this review (see PRISMA flow chart in Appendix B for details). Only two sources were identified for inclusion, both of which were found in the grey literature search. No academic publications met the criteria for extraction in this review. As such, the critical finding in this review is the significant lack of available policy-related materials providing guidance for the prevention, early intervention, and treatment of EDs for FNMI populations in Canada.

From the four databases used in the academic literature search, 541 references were imported to Covidence for screening. A total of 86 duplicates were automatically identified by Covidence and 455 publications were screened for title and abstract relevance. Of the 455 publications, seven were advanced to be screened at full text, of which three were excluded due to their focus on a non-Indigenous population, one due to unavailability of full text, one due to it having been conducted in the USA, one that was published before 2015 and the TRC’s Calls to Action, and one due to a lack of relevance to policy. No publication met all the inclusion criteria to be advanced to the extraction stage. As such, the major findings from the database search are rather the lack thereof.

We screened 736 grey literature websites, including but not limited to FNMI-led organizations (e.g., First Nations health authorities, Tribal Councils, etc.), Canadian national and provincial/territorial ED and mental health organizations, and medical authorities across the country. From these websites, only two documents met the inclusion criteria for extraction. Screening of the 638 First Nations communities listed by the Government of Canada (Government of Canada, n.d.) yielded little by way of policy documents, though four communities’ websites made specific, albeit brief, mention of EDs among their mental health service provisions. Many communities made explicit references to diabetes and nutritional initiatives and programming, although this did not meet the inclusion criteria.

Two eligible grey literature documents were published between 2023 and 2025 by the National Eating Disorder Information Centre (NEDIC) in Canada. Both were authored by individuals with lived experience of EDs, and both included more expansive definitions of EDs, referring to both EDs specifically, and disordered eating more generally. The first, a commentary titled “Nourishing our spirits: radical love and sovereign healing,” by Parker and Wilcox (2025), is a call to action oriented toward policymakers and practitioners at all system levels. It calls for the acknowledgement of the continuing and disproportionate harm perpetuated by historical and ongoing colonialism resulting in the disruption of traditional food practices and relationships with land and food. The commentary explains that Westernized treatments in EDs do not include Indigenous perspectives, and as such lack contextually important considerations. It references blood memory among these considerations, wherein individuals are connected to ancestors from whom they draw on strength and resilience. Finally, this article emphasizes the critical recognition of intergenerational trauma, nutritional experiments, and medical neglect that contribute to lower access to safe and culturally relevant care. The authors call on practitioners to educate themselves on intergenerational trauma, cultural knowledge, and consider historical and present-day contexts that may impact Indigenous Peoples with EDs and the prevention and early intervention of EDs (Parker & Wilcox, 2025).

The second document, an educational resource by Miskonoodinkwe-Smith et al. (2023), was positioned as a supportive resource for individuals with an ED or disordered eating, their families and loved ones. This document discussed contributing factors of EDs and disordered eating, such as social comparison, while also describing the colonial impact on these contributing factors, including a lack of education on EDs and Westernized beauty standards. The authors encourage consideration of traditional healing methods, inclusion of family and friends in treatment, and acknowledgement of barriers that Indigenous Peoples may face when seeking treatment (Miskonoodinkwe-Smith et al., 2023). Across both Parker and Wilcox’s (2025) and Miskonoodinkwe-Smith et al.’s (2023) documents, the importance and awareness of the historical and present colonial contexts of FNMI Peoples and cultural considerations in treatment were emphasized.

## 4. Discussion

This policy scoping review found only two documents informing the prevention, early intervention, and treatment of EDs for FNMI populations in Canada, none of which were sourced from the academic literature. Both documents, one a call to action and the second an educational resource for individuals with lived experience or their loved ones, provide important insights. Notably, both were produced by NEDIC, the leading Canadian organization in ED education and advocacy. Their authorship illustrates that national attention to Indigenous experiences of EDs is currently concentrated within NEDIC’s outreach and resource development efforts, signaling growing institutional awareness of the need for culturally grounded approaches and the importance of collaborating with individuals and communities with lived experience to ensure relevance and authenticity in future policy and practice development. However, no formal policy documentation was found. Given this absence, health and wellness organizations, along with federal, provincial, and territorial governments in Canada, have an opportunity to assess if their guiding policies support all patient populations they serve.

This review mapped existing policy documentation for EDs within FNMI populations. Given the infancy of this field, we anticipated limited publications meeting the criteria, though we were surprised that the search of the academic literature produced no results. Our study team debated further expansion of the inclusion criteria but could not reasonably consider methodological amendments to make our search any broader. The absence of ED policies specific to FNMI is not simply a gap in documentation but a structural barrier that limits prevention, early identification, and culturally and contextually grounded care. It is worth noting that there is an absence of research using a distinctions-based approach in FNMI populations for ED policies. There is growing awareness that EDs negatively impact FNMI Peoples (Alani-Verjee et al., 2017; Emelianchik-Key et al., 2023; Mohammed et al., 2026); however, there is a lack of research that supports rural and urban communities’ knowledge on this growing concern. The continued absence of prevalence rates and epidemiological data renders FNMI populations aggregated into broader Indigenous or mainstream populations regarding ED prevention, early intervention, and treatment. ED policy and future practice guidelines must consider the unique colonial and cultural considerations in the development, implementation, and evaluation of services for FNMI Peoples.

The integration of blood memory was reflected in one document in this review (Parker & Wilcox, 2025). Parker and Wilcox (2025) defined blood memory as an intergenerational transmission of stories, traits, and various abilities and behaviors passed from parents to children. FNMI Peoples experienced various famines and traumas (Daschuk, 2013; Mosby, 2013), and the effects of widespread famine and malnutrition on previous generations may have an impact on future generations through epigenetic mechanisms (Yehuda & Lehrner, 2018). Brewerton et al. (2022) note that adverse childhood experiences, including historical trauma (e.g., epigenetics), can help explain the impact of various traumas on individuals with EDs. Understanding historical trauma and the implication of epigenetics is salient to inform treatment practice (Gill et al., 2026) and trauma-informed care (Marcellus & Ringham, 2025). Trauma can present in various forms, including intergenerationally, and social supports and access to resources can impact how an individual is affected (Marcellus & Ringham, 2025). Trauma-informed care should include the cultural and colonial contexts of individuals, and value culturally relevant and responsive relationships (Marcellus & Ringham, 2025).

Common negative and deficit-based narratives that describe FNMI Peoples have generated shame and stigmatization (Hyett et al., 2019). The shift toward a strengths-based approach is reflected by mental healthcare and research with FNMI Peoples increasingly recognizing their capabilities and resiliency (Bryant et al., 2021). The literature on clinicians’ attitudes toward individuals with EDs suggests that some may hold clients responsible for their EDs, overlooking situations beyond the client’s control (Thompson-Brenner et al., 2012). Relatedly, when future health professionals learn about historical and ongoing colonialism, they often attribute the causes of poor health and social outcomes to FNMI Peoples, rather than to structural determinants of health (Melro et al., 2023). Taken together, these findings highlight the challenges of improving the prevention, early intervention, and treatment of EDs with FNMI Peoples, and underscore the importance of integrating lived realities and perspectives within ED service planning and education of future and practicing health professionals. Too often, treatment and assessment overlook the historical and ongoing colonial and structural determinants of health, such as the intergenerational impact of residential school experiences and disruptions to traditional food practices. Shifting the narrative to a strengths-based approach in ED care acknowledges the perseverance and wisdom FNMI people with EDs have (Plante et al., 2026). One way to bolster health among FNMI Peoples may be through the use of language to connect to one’s culture (Whalen et al., 2016). With relevance to this review and the work being done in the ED landscape, the NEDIC’s ‘Guide for Family’ is available in Inuktitut, demonstrating a gradual shift toward providing accessible resources to reduce barriers and support language revitalization (National Initiative for Eating Disorders, 2022). This shift toward language revitalization is in alignment with Māori, who have created a language glossary for EDs (Fraser et al., 2024). Understanding culturally relevant constructs may provide an opportunity to integrate strengths-based approaches into treatment.

Practitioners are encouraged to integrate FNMI perspectives that fall outside Western-based conceptualizations of EDs into their practice. As evidenced globally, the integration of FNMI perspectives is critical for developing best practices for supporting FNMI individuals with EDs because it supports a growing understanding of cultural views, traditions, and practices that support healing (Smith et al., 2021). We acknowledge that non-FNMI practitioners may lack training opportunities to expand and integrate their knowledge into practice. Although the field is ripe with a reiteration of calling for the development and integration of culturally safe practices, we recommend that the ED field meaningfully, respectfully, and humbly work with rural and urban FNMI communities to educate and work collaboratively to support FNMI People with, or at risk of, EDs.

### Limitations and Areas for Future Study

In presenting the findings of this review, we could not take a distinctions-based approach due to a lack of disaggregated data in the included material. Through our expansive academic and grey literature search, we sought to comprehensively capture what exists for FNMI-specific policy and practices in Canada. Despite this extensive search, nothing was found within the peer-reviewed literature, limiting the scope of our analysis, though this likely reflects the infancy of this field of work rather than the expanse of the search. In future work, we aim to mitigate these limitations by calling for the development of the academic and grey literature building upon ED policy relevant to FNMI populations in Canada, with particular emphasis on each community’s unique, distinct contexts and needs. Similarly, given the broader inclusion of EDs and disordered eating in both articles present in this review, it may be prudent to expand our definition of EDs to better capture disordered eating in future work. With the limited literature currently available relevant to FNMI populations, and the present underreporting of EDs, a more permissive definition may make future work more relevant and accessible to a broader audience.

For future work emerging from and informed by this review, we will invest more time and effort into community engagement in the research process. With this foundational understanding of what is and is not presently available, community engagement becomes a primary focus of future work. The present study is further limited in this regard, particularly because the only documents that could be included were those that could be found either online or in the academic databases included in the search. We imagine that other policies and programs may exist within communities that may not be publicly available online, and further engagement with said communities in the future could help bring these to light. We recognize the burden placed on communities associated with garnering this knowledge and developing better practices for culturally sound ED policy development. As such, a collaborative approach bolstered by recognition and funding by the government is critical. We endeavor to conduct future work across policy, program, and practice levels to better understand and address EDs for and with FNMI populations going forward, and encourage the wider ED field within Canada to prioritize the same.

## 5. Conclusions

This scoping review reveals a profound absence of ED policy-related documentation relevant to First Nations, Inuit, and Métis Peoples. The findings were limited to two documents: a commentary and an educational resource. Despite the limited results, the findings point to a clear need for policy action. The included resources call for service providers to integrate and adopt willingness and humility to integrate FNMI knowledge into care. These findings align with broader evidence showing that FNMI mental health needs are often rendered invisible within mainstream policy frameworks, reinforcing the urgency of distinctions-based, community-driven approaches. As the Canadian practice guidelines for the treatment of EDs are undergoing an update, it will be prudent to include calls for cultural considerations for the prevention, early intervention, and treatment of EDs. Addressing these gaps requires meaningful partnership with FNMI communities and a commitment to distinctions-based, culturally grounded approaches that honor FNMI knowledge systems and support the wellbeing of FNMI individuals seeking care for EDs in Canada.

## Figures and Tables

**Table 1:** Grey literature sources.

Source Type	Count
Health Organizations	
Canadian Eating Disorder Organization	20
Canadian Health Centre or Organization	12
Provincial/Territorial Health Organization	6
Youth Wellness Organization/IYS Hub	8
Mental Health Organization	7
FNMI Nations/Organizations	
First Nations	638
FNMI Health Centre or Organization	27
Other FNMI Organization (i.e., Councils, Inuit Hamlets)	11
Métis Organizations	7

## Data Availability

We have made our tracking documentation (i.e., website list, screening decisions, extracted data, and reasons for full-text exclusion) available within the manuscript’s Supplementary Materials. For additional information, please reach out to the corresponding author.

## Supplementary Materials

The following supporting information can be downloaded at: https://www.mdpi.com/article/10.3390/bs16091558/s1, File S1: Preferred Reporting Items for Systematic reviews and Meta-Analyses extension for Scoping Reviews (PRISMA-ScR) Checklist; File S2: Grey literature screening and extraction.

## Abbreviations

The following abbreviations are used in this manuscript:

ED	Eating Disorder
FNMI	First Nations, Métis, and Inuit
BIPOC	Black, Indigenous, and People of Color
TRCC	Truth and Reconciliation Commission of Canada
NEDIC	National Eating Disorder Information Centre

## Appendix A. Academic Literature Search Strategy

Database	Concept: Eating disorders and disordered eating	Concept: Indigenous Peoples in Canada
Bibliography of Indigenous Peoples in North America	(anorex* OR bulim* OR “binge* eat*” OR purg*) OR ((eat* OR feed* OR food OR appetite OR purg*) n3 disorder*) OR (“Avoidant Restrictive Food Intake Disorder” OR OSFED OR ARFID)	aboriginal* OR amerind* OR autochtone* OR “first nation*” OR indigenous* OR indigenist* OR inuit* OR eskimo* OR inupiat* OR metis OR “native canadian*” OR “native people*” OR “First People” OR “Status Indian” OR algonquin OR anishinabek OR anishnabek OR chipewyan OR “cree” OR “dene” OR gitksan OR haudenosaunee OR huron OR “innu” OR inuktitut OR inuk OR iqaluit OR iroquois OR kawawachikamach OR kahnawake OR kitikmeot O “mi’khaki” OR “mi’kmaq” OR “mi’kmaw” OR mohawk OR mushkegowuk OR naskapi OR “nisga’a” OR “oji- cree” OR ojibway OR “oki” OR opaskwayak OR pauktuutit OR qikiqtani OR qayuqtuvik OR “rankin inlet” OR “sioux” OR tungasugit OR tuttarvingat OR “vuntut gwitchin” OR akwesasne OR athabascan OR “canadian arctic” OR “chesterfield inlet” OR “northwest territories” OR “eeyou istchee” OR inukjuak OR igluligaarjuk OR inuvialuit OR ivujivik OR “james bay” OR kuujjuaq OR “mackenzie river basin” OR mistissini OR nemaska OR nunatsiavut OR nunavut OR nunavik OR nutaqqavut OR “ouje-bougoumou” OR shubenacadie OR “slave lake” OR “subarctic ontario” OR wikwemikong OR Blackfoot OR Kainai OR Anishnawbe OR “An Act respecting Indians” OR “Indian Act” OR “Status Indian” OR “Non-Insured Health Benefit*” OR “NIHB”
CINAHL	(MH “Eating Disorders+”) OR ((anorex* OR bulim* OR “binge* eat*” OR purg*) OR ((eat* OR feed* OR food OR appetite OR purg*) n3 disorder*) OR (“Avoidant Restrictive Food Intake Disorder” OR OSFED OR ARFID))	(MH “Indigenous Peoples+” OR MH “Aboriginal Canadians+”) OR (aboriginal* OR amerind* OR autochtone* OR “first nation*” OR indigenous* OR indigenist* OR inuit* OR eskimo* OR inupiat* OR metis OR “native canadian*” OR “native people*” OR “First People” OR “Status Indian” OR algonquin OR anishinabek OR anishnabek OR chipewyan OR “cree” OR “dene” OR gitksan OR haudenosaunee OR huron OR “innu” OR inuktitut OR inuk OR iqaluit OR iroquois OR kawawachikamach OR kahnawake OR kitikmeot O “mi’khaki” OR “mi’kmaq” OR “mi’kmaw” OR mohawk OR mushkegowuk OR naskapi OR “nisga’a” OR “oji- cree” OR ojibway OR “oki” OR opaskwayak OR pauktuutit OR qikiqtani OR qayuqtuvik OR “rankin inlet” OR “sioux” OR tungasugit OR tuttarvingat OR “vuntut gwitchin” OR akwesasne OR athabascan OR “canadian arctic” OR “chesterfield inlet” OR “northwest territories” OR “eeyou istchee” OR inukjuak OR igluligaarjuk OR inuvialuit OR ivujivik OR “james bay” OR kuujjuaq OR “mackenzie river basin” OR mistissini OR nemaska OR nunatsiavut OR nunavut OR nunavik OR nutaqqavut OR “ouje-bougoumou” OR shubenacadie OR “slave lake” OR “subarctic ontario” OR wikwemikong OR Blackfoot OR Kainai OR Anishnawbe OR “An Act respecting Indians” OR “Indian Act” OR “Status Indian” OR “Non-Insured Health Benefit*” OR “NIHB”)
PubMed (MedLine)	((“feeding and eating disorders”[MeSH Major Topic] AND 1000/01/01:2026/05/01[Date—Publication]) OR ((“anorex*”[All Fields] OR “bulim*”[All Fields] OR “binge eat”[All Fields] OR “binge eating”[All Fields] OR “purg*”[All Fields]) AND 1000/01/01:2026/05/01[Date—Publication]) OR (“eating disorder”[Title/Abstract:3] AND 1000/01/01:2026/05/01[Date—Publication]) OR (“disordered eating”[Title/Abstract:3] AND 1000/01/01:2026/05/01[Date—Publication]) OR (“feeding disorders”[Title/Abstract:3] AND 1000/01/01:2026/05/01[Date—Publication]) OR (“food disorder”[Title/Abstract:3] AND 1000/01/01:2026/05/01[Date—Publication]) OR (“appetite disorder”[Title/Abstract:3] AND 1000/01/01:2026/05/01[Date—Publication]) OR (“purging disorder”[Title/Abstract:3] AND 1000/01/01:2026/05/01[Date—Publication]) OR (“Avoidant Restrictive Food Intake Disorder”[All Fields] AND 1000/01/01:2026/05/01[Date—Publication]) OR (“ARFID”[All Fields] AND 1000/01/01:2026/05/01[Date—Publication]) OR (“Other Specified Feeding or Eating Disorder”[All Fields] AND 1000/01/01:2026/05/01[Date—Publication]) OR (“OSFED”[All Fields] AND 1000/01/01:2026/05/01[Date—Publication])) AND (1000/1/1:2026/5/1[pdat])	((“indigenous canadians”[MeSH Major Topic] AND 1000/01/01:2026/05/01[Date—Publication]) OR ((“aboriginal*”[All Fields] OR “amerind*”[All Fields] OR “autochtone*”[All Fields] OR “first nation*”[All Fields] OR “indigenous*”[All Fields] OR “indigenist*”[All Fields] OR “inuit*”[All Fields] OR “eskimo*”[All Fields] OR “inupiat*”[All Fields] OR “metis”[All Fields] OR “native canadian*”[All Fields] OR “native people*”[All Fields] OR “first people*”[All Fields] OR “Status Indian”[All Fields] OR “algonquin”[All Fields] OR “anishinabek”[All Fields] OR “anishnabek”[All Fields] OR “chipewyan”[All Fields] OR “cree”[All Fields] OR “dene”[All Fields] OR “gitksan”[All Fields] OR “haudenosaunee”[All Fields] OR (“huron”[All Fields] OR “huron s”[All Fields]) OR “innu”[All Fields] OR “inuktitut”[All Fields] OR (“inuit”[MeSH Terms] OR “inuit”[All Fields] OR “inuk”[All Fields]) OR “iqaluit”[All Fields] OR “iroquois”[All Fields] OR “kawawachikamach”[All Fields] OR “kahnawake”[All Fields] OR “kitikmeot”[All Fields] OR “kitimat”[All Fields] OR “kivalliq”[All Fields] OR (“kwakiutl indians”[Supplementary Concept] OR “kwakiutl indians”[All Fields] OR “kwakiutl”[All Fields]) OR “manitoulin”[All Fields] OR “miawpukek”[All Fields] OR “micmac”[All Fields] OR “mi’kmaq”[All Fields] OR “mi’kmaw”[All Fields] OR (“mohawk”[All Fields] OR “mohawks”[All Fields]) OR “mushkegowuk”[All Fields] OR “naskapi”[All Fields] OR “nisga’a”[All Fields] OR “oji-cree”[All Fields] OR “ojibway”[All Fields] OR “oki”[All Fields] OR “opaskwayak”[All Fields] OR “pauktuutit”[All Fields] OR “qikiqtani”[All Fields] OR “rankin inlet”[All Fields] OR “sioux”[All Fields] OR “vuntut gwitchin”[All Fields] OR “akwesasne”[All Fields] OR (“athabascan”[All Fields] OR “athabascans”[All Fields]) OR “canadian arctic”[All Fields] OR “chesterfield inlet”[All Fields] OR “northwest territories”[All Fields] OR “eeyou istchee”[All Fields] OR “inukjuak”[All Fields] OR (“mackenzie eskimos”[Supplementary Concept] OR “mackenzie eskimos”[All Fields] OR “inuvialuit”[All Fields]) OR “ivujivik”[All Fields] OR “james bay”[All Fields] OR “kuujjuaq”[All Fields] OR “mackenzie river basin”[All Fields] OR “mistissini”[All Fields] OR “nemaska”[All Fields] OR “nunatsiavut”[All Fields] OR (“nunavut”[MeSH Terms] OR “nunavut”[All Fields]) OR “nunavik”[All Fields] OR “nutaqqavut”[All Fields] OR “ouje-bougoumou”[All Fields] OR “shubenacadie”[All Fields] OR “slave lake”[All Fields] OR “subarctic ontario”[All Fields] OR “wikwemikong”[All Fields] OR “Blackfoot”[All Fields] OR “Kainai”[All Fields] OR “Anishnawbe”[All Fields] OR (“act”[All Fields] AND (“respect”[MeSH Terms] OR “respect”[All Fields] OR “respected”[All Fields] OR “respects”[All Fields] OR “respectability”[All Fields] OR “respectable”[All Fields] OR “respectful”[All Fields] OR “respectfulness”[All Fields] OR “respecting”[All Fields] OR “respective”[All Fields] OR “respectively”[All Fields]) AND (“indian”[All Fields] OR “indian s”[All Fields] OR “indians”[All Fields])) OR “Indian Act”[All Fields] OR “status indian*”[All Fields] OR “non insured health benefit*”[All Fields] OR “NIHB”[All Fields]) AND 1000/01/01:2026/05/01[Date—Publication])) AND (1000/1/1:2026/5/1[pdat])
PsycINFO (ProQuest)	MAINSUBJECT.EXACT.EXPLODE(“Eating Disorders”) OR (anorex* OR bulim* OR “binge* eat*” OR purg*) OR ((eat* OR feed* OR food OR appetite OR purg*) n3 disorder*) OR (“Avoidant Restrictive Food Intake Disorder” OR OSFED OR ARFID)	MAINSUBJECT.EXACT.EXPLODE (“Indigenous Peoples in Canada”) OR aboriginal* OR amerind* OR autochtone* OR “first nation*” OR indigenous* OR indigenist* OR inuit* OR eskimo* OR inupiat* OR metis OR “native canadian*” OR “native people*” OR “First People*” OR “Status Indian” OR algonquin OR anishin abek OR anishnabek OR chipewy an OR “cree” OR “dene” OR gitksan OR haudenosaunee OR huron OR “innu” OR inuktitut OR inuk OR iqaluit OR iroquois OR kawawachikamac h OR kahnawake OR kitikmeot OR kitimat OR kivalliq OR kwakiutl OR manitoulin OR miawpukek O R micmac OR “mi’khaki” OR “mi’kmaq” OR “mi’kmaw” OR mohawk OR mushkegowuk OR naskapi OR “nisga’a” OR “oji-cree” OR ojibway OR “oki” OR opaskw ayak OR pauktuutit OR qikiqtani OR qayuqtuvik OR “rankin inlet” OR “sioux” OR tungasugit OR tutt arvingat OR “vuntut gwitchin” OR akwesasne OR athabascan OR “canadian arctic” OR “chesterfield inlet” OR “northwest territories” OR “eeyou istchee” OR inukjuak OR igluligaarjuk OR i nuvialuit OR ivujivik OR “james bay” OR kuujjuaq OR “mackenzie river basin” OR mistissini OR nemaska OR nunatsiavut OR nunavut OR nunavik OR nutaqqavut OR “ouje-bougoumou” OR shubenacadie OR “slave lake” OR “subarctic ontario” OR wikwemikong OR Blackfoot OR Kainai OR Anishnawbe OR “An Act respecting Indians” OR “Indian Act” OR “Status Indian” OR “Non-Insured Health Benefit” OR “NIHB”
